# Correction: Antigen-presenting autoreactive B cells activate regulatory T cells and suppress autoimmune arthritis in mice

**DOI:** 10.1084/jem.2023010109212023c

**Published:** 2023-10-03

**Authors:** Mike Aoun, Ana Coelho, Alexander Krämer, Amit Saxena, Pierre Sabatier, Christian Michel Beusch, Erik Lönnblom, Manman Geng, Nhu-Nguyen Do, Zhongwei Xu, Jingdian Zhang, Yibo He, Laura Romero Castillo, Hassan Abolhassani, Bingze Xu, Johan Viljanen, Joanna Rorbach, Gonzalo Fernandez Lahore, Inger Gjertsson, Alf Kastbom, Christopher Sjöwall, Jan Kihlberg, Roman A. Zubarev, Harald Burkhardt, Rikard Holmdahl

Vol. 220, No. 11 | https://doi.org/10.1084/jem.20230101 | September 11, 2023

In the originally published article, the figure images for Figs. 4 and S4 were accidentally swapped with Figs. 5 and S5, respectively; the legends remain unchanged. The corrected figures and their legends are shown here.

In addition, figure citations and a typographical error were corrected in the Results and Materials and methods. Changes are indicated with underlined text as follows:

## Results

### Antigen-presenting C1-B induce COL2-specific Treg proliferation in a contact-dependent manner

Despite the ability of C1-B in secreting IL10 (Fig. S3 C), we failed to detect a prominent proliferation of Tregs in the contactless set-up.

### C1-B hold an antigen presentation signature and upregulate CCR7 and CD72 upon activation

While we showed that autoreactive C1-B exist in mice and men and on the transcriptional level mouse and human gene profiles are overall conserved, we chose to define the more relevant C1-B from HD by transcriptomics to later validate the hits in the mouse.

To better delineate the C1-B population, we immunophenotyped LN-derived cells from BQ mice 30 d after COL2 immunization (Fig. 5 K).

## Materials and methods

### Serum and cartilage samples

Serum samples, used in Fig. S1 F, from three different cohorts were employed.

These changes do not alter the conclusions of the manuscript. The errors appear in PDFs downloaded before September 20, 2023.

**Figure 4.**
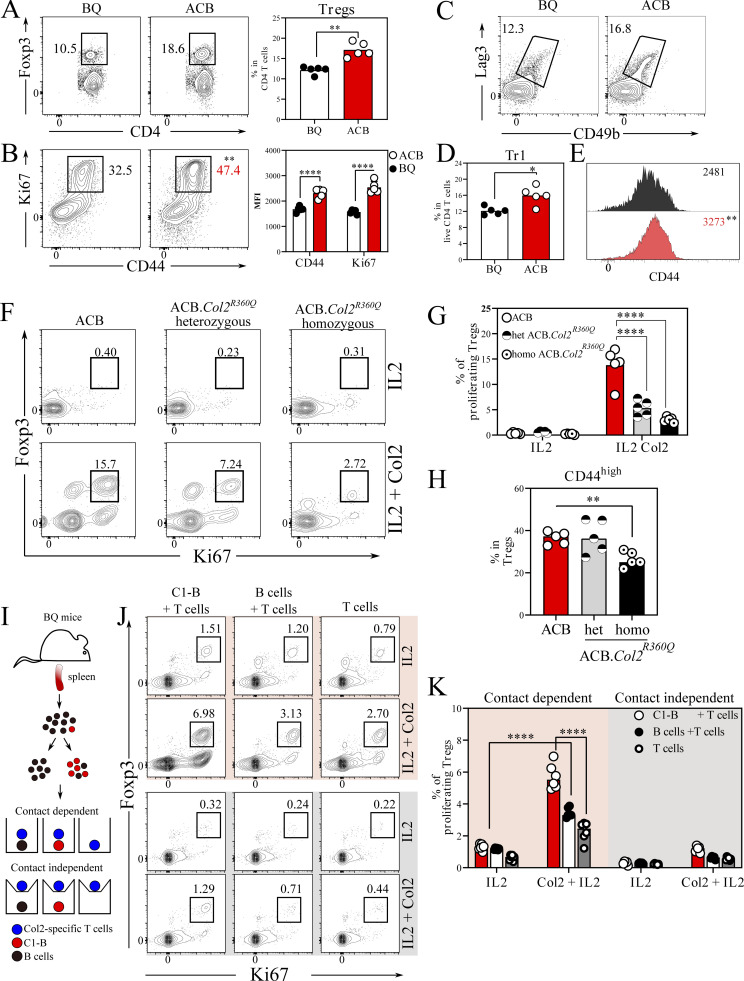
**C1-B are potent inducers of Tregs. (A and B)** Frequency, activation, and proliferation of FOXP3^+^ Tregs in the LNs of 10 d immunized ACB and BQ mice (*n* = 5 mice/group). **(C–E)** Frequency and activation of LAG3^+^CD49b^+^ Tr1 cells in the LNs of 10 d immunized ACB and BQ. **(F and G)** Flow cytometry plots depicting the frequency of endogenous COL2-reactive Vβ8.3 proliferating Tregs (FOXP3^+^Ki67^+^) after in vitro culture of LN cells derived from ACB, heterozygous and homozygous ACB.*Col2*^*R360Q*^ mice (*n* = 5 mice/group). **(H)** Frequency of CD44^+^ Tregs gated on F. **(I)** Experimental setup for contact-dependent and independent Treg induction experiment. **(J and K)** Flow cytometry plots depicting the frequency of proliferating Tregs in direct contact (pink), contactless (gray) culture alone or with natural C1-B and B cells purified from BQ mice in the presence or absence of COL2 (*n* = 18 mice, each symbol represents three mice pooled). Error bars represent mean ± SEM. Statistics in A, B, D, and E were determined by two-tailed Mann–Whitney *U* test. Significance in G, H, and K was determined by two-way ANOVA followed by Sidak’s test. ****P < 0.0001.

**Figure 5.**
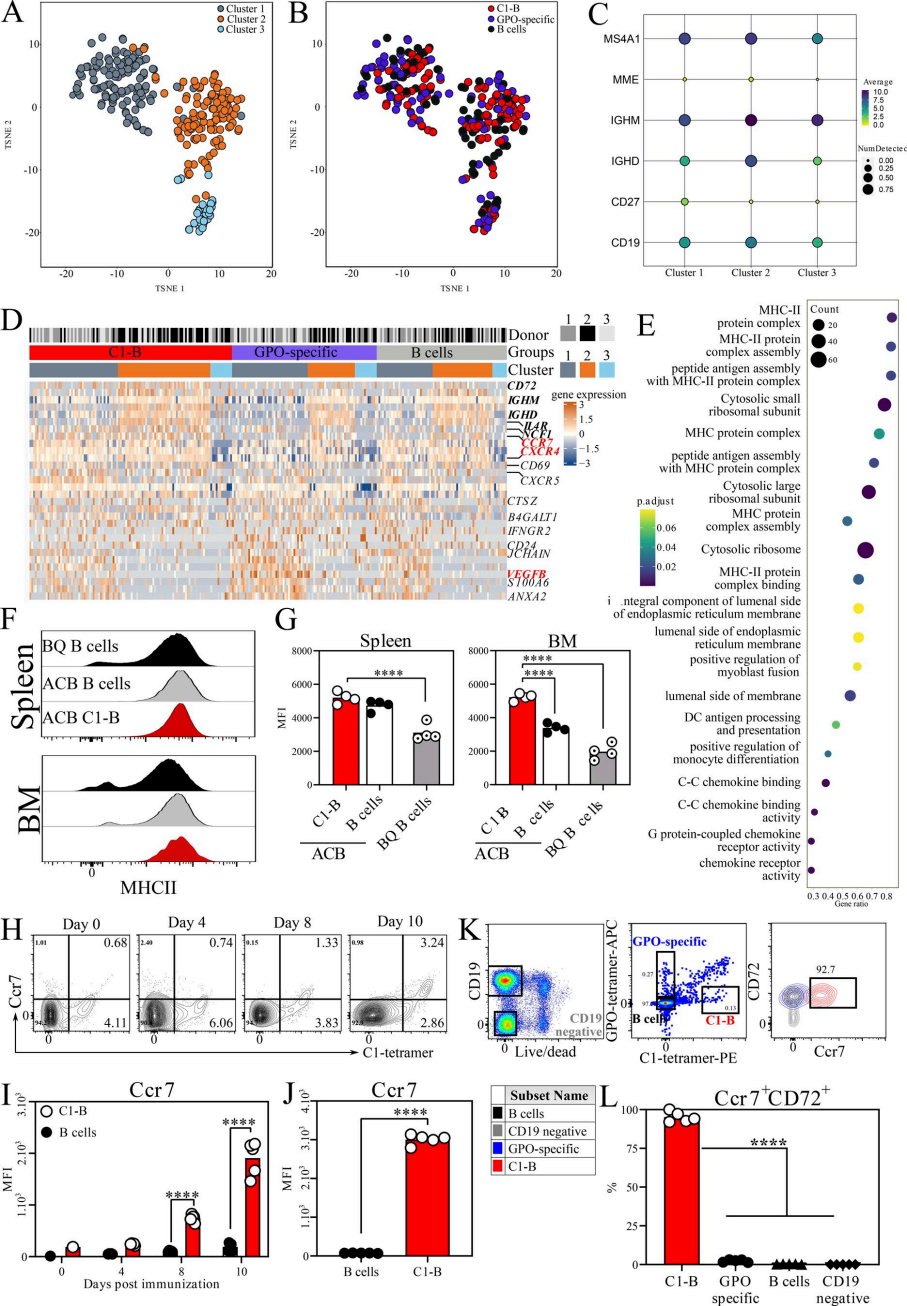
**Antigen-presenting C1-B upregulates CCR7 and CD72 upon activation. (A)** RNA sequencing of single sorted C1-B, GPO-specific, and B cells from HD (*n* = 3). Data presented as t-SNE. **(B)** The distribution of different groups within the clusters. **(C)** The expression of B cell subtype markers where a dot represents each feature (row) in each group of cells (column). The proportion of detected expression values and the average expression for each feature in each group of cells is visualized using the size and color, respectively, of each dot. **(D)** Heatmap of selected immune-related genes that are significantly differentially expressed (in red) and belong to the top 50 differentially regulated genes but not statistically significant (in bold black). **(E)** The summary of top enriched terms between C1-B and GPO-specific B cells depicting the enrichment score, gene ratio, and gene count. **(F and G)** Representative flow cytometry plots and mean fluorescent intensity (MFI) quantification depicting surface MHCII expression on BM- and spleen-derived B cells from ACB and BQ mice (*n* = 4/group). **(H and I)** Frequency and MFI of CCR7 on circulating C1-B days after ACB immunization. **(J)** CCR7 MFI on natural C1-B derived from LN of 10 d immunized BQ mice. **(K and L)** LN from day 30 immunized BQ mice were stained ex vivo and analysis of CD72 and CCR7 was assessed on C1-B (red), GPO-specific (blue), B cells (black), and non-B cells (gray). Error bars represent mean ± SEM. Statistics in J were determined by two-tailed Mann–Whitney *U* test. Significance in I and L was determined by two-way ANOVA followed by Sidak’s test. **P < 0.01 and ****P < 0.0001.

**Figure S4.**
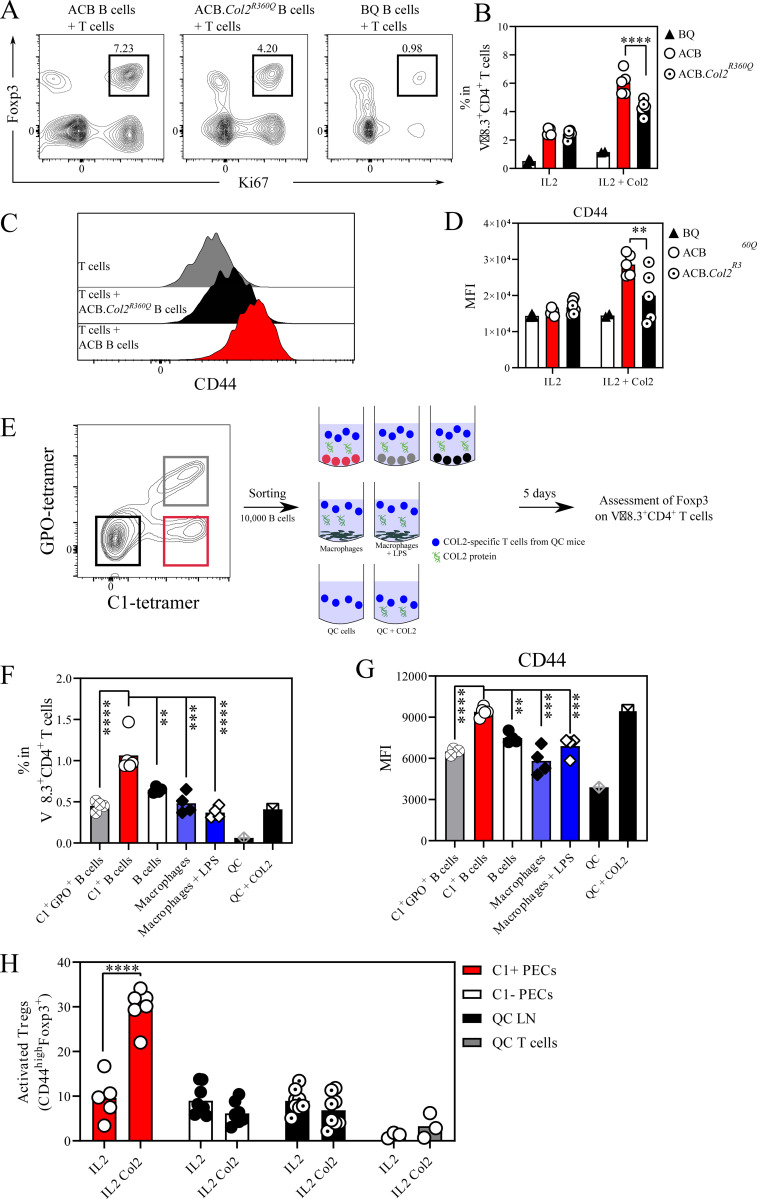
**C1-B controls the expansion of Tregs in vitro. (A)** Representative flow cytometry plot of the Foxp3^+^Ki67^+^ gating 48 h after coculture of sorted QC T cells with sorted B cells from ACB, ACB.*Col2*^*360Q*^, or BQ WT mice in the presence or absence of COL2. **(B)** Frequency of proliferating Tregs within the Vβ8.3^+^CD4^+^ T cell population. **(C)** Representative histograms exhibiting the expression of CD44 on proliferating Tregs. **(D)** Quantification of CD44 MFI on proliferating Tregs from A. **(E)** Gating strategy and experimental design of the sorted 10,000 C1-B, C1-GPO-specific, and naïve B cells cocultured with sorted QC T cells for 5 d in the presence of COL2. **(F)** Frequency of Foxp3^+^ T cells within the Vβ8.3^+^CD4^+^ population. **(G)** Quantification of CD44 MFI on Tregs from F. **(H)** Frequency of activated Tregs within the Vβ8.3^+^CD4^+^ T cell population following coculture with C1-B (C1^+^), C1-B depleted (C1^−^), or without (total QC LN or isolated QC T cells alone) in the presence or absence of COL2. Error bars represent mean ± SEM. Statistical significance in B and D was determined using two-tailed Mann–Whitney *U* test while in F–H was determined using one-way ANOVA followed by Sidak’s correction test. **P < 0.01 and ***P < 0.001.

**Figure S5.**
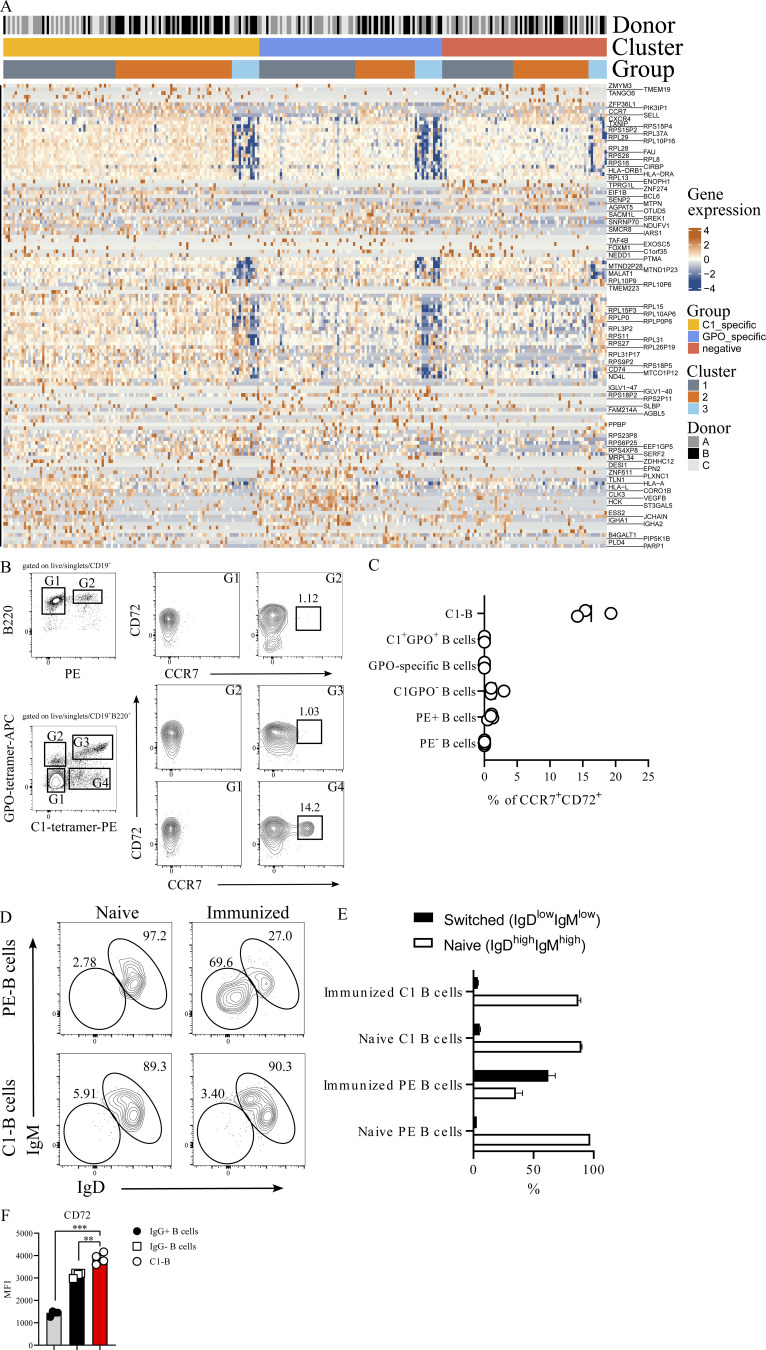
**CCR7/CD72 expression is restricted to autoreactive C1-B. (A)** Heatmap of the genes that are significantly expressed grouped according to the three donors (A, B, and C), the three B cell population groups (C1-specific, GPO-specific, and double negative B cells), and the three clusters (cluster 1 = memory cells, cluster 2 = naïve cells, cluster 3 = B1-like). Normalized log counts are plotted after scaling by row for the top 50 markers (Wilcoxon rank sum test, scran). **(B)** Enriched PE-specific B cells and C1-B from spleens and iLN of BQ mice 10 d after immunization with PE and Col2, respectively. Naïve mice were also included. Representative flow cytometry plots exhibiting the surface expression of CD72 and CCR7 on different gated (G) populations. **(C)** Dot plot quantifying the frequency of CCR7/CD72 double-positive cells within the different gated populations. **(D)** Representative flow cytometry plots exhibiting IgM and IgD surface expression on PE- and C1- B cells enriched from naïve and 10 d immunized BQ mice. **(E)** Bar plot quantifying the frequency of naïve (IgD^high^/IgM^high^) and switched (IgD^low^/IgM^low^) C1- and PE-specific B cells enriched from naïve (*n* = 1–2/group) and 10 d immunized (*n* = 3/group) BQ mice. **(F)** MFI of surface CD72 on enriched C1-B, IgG^−^, and IgG^+^ B cells isolated from HD PBMC (*n* = 4). Error bars represent mean ± SEM. Statistical significance in F was determined using one-way ANOVA followed by Sidak’s correction test. **P < 0.01, ***P < 0.001, and ****P < 0.0001.

